# MicroRNA expression analysis in peripheral blood and soft-tissue of patients with periprosthetic hip infection: a prospective controlled pilot study

**DOI:** 10.1302/2633-1462.56.BJO-2023-0172.R2

**Published:** 2024-06-06

**Authors:** Alp Paksoy, Sebastian Meller, Florian Schwotzer, Philipp Moroder, Andrej Trampuz, Jan-Philipp Imiolczyk, Carsten Perka, Matthias Hackl, Fabian Plachel, Doruk Akgün

**Affiliations:** 1 Charité University Hospital, Center for Musculoskeletal Surgery, Berlin, Germany; 2 Schulthess Klinik, Zurich, Switzerland; 3 TAmiRNA GmbH, Vienna, Austria; 4 Healthlab, Salzburg, Austria

**Keywords:** Biomarker, Circulating microRNA, Periprosthetic hip joint infection, MicroRNA signature, CRP, MicroRNA expression level, MicroRNA profiling

## Abstract

**Aims:**

Current diagnostic tools are not always able to effectively identify periprosthetic joint infections (PJIs). Recent studies suggest that circulating microRNAs (miRNAs) undergo changes under pathological conditions such as infection. The aim of this study was to analyze miRNA expression in hip arthroplasty PJI patients.

**Methods:**

This was a prospective pilot study, including 24 patients divided into three groups, with eight patients each undergoing revision of their hip arthroplasty due to aseptic reasons, and low- and high-grade PJI, respectively. The number of intraoperative samples and the incidence of positive cultures were recorded for each patient. Additionally, venous blood samples and periarticular tissue samples were collected from each patient to determine miRNA expressions between the groups. MiRNA screening was performed by small RNA-sequencing using the miRNA next generation sequencing (NGS) discovery (miND) pipeline.

**Results:**

Overall, several miRNAs in plasma and tissue were identified to be progressively deregulated according to ongoing PJI. When comparing the plasma samples, patients with a high-grade infection showed significantly higher expression levels for hsa-miR-21-3p, hsa-miR-1290, and hsa-miR-4488, and lower expression levels for hsa-miR-130a-3p and hsa-miR-451a compared to the aseptic group. Furthermore, the high-grade group showed a significantly higher regulated expression level of hsa-miR-1260a and lower expression levels for hsa-miR-26a-5p, hsa-miR-26b-5p, hsa-miR-148b-5p, hsa-miR-301a-3p, hsa-miR-451a, and hsa-miR-454-3p compared to the low-grade group. No significant differences were found between the low-grade and aseptic groups. When comparing the tissue samples, the high-grade group showed significantly higher expression levels for 23 different miRNAs and lower expression levels for hsa-miR-2110 and hsa-miR-3200-3p compared to the aseptic group. No significant differences were found in miRNA expression between the high- and low-grade groups, as well as between the low-grade and aseptic groups.

**Conclusion:**

With this prospective pilot study, we were able to identify a circulating miRNA signature correlating with high-grade PJI compared to aseptic patients undergoing hip arthroplasty revision. Our data contribute to establishing miRNA signatures as potential novel diagnostic and prognostic biomarkers for PJI.

Cite this article: *Bone Jt Open* 2024;5(6):479–488.

## Introduction

Periprosthetic joint infection (PJI) is one of the most devastating complications after hip arthroplasty,^1^ and is commonly treated surgically by debridement, antibiotics and implant retention (DAIR), and single- or multistage revision.^2,3^ With rising numbers of arthroplasties being performed, and as a result of the growing elderly population, a subsequent increase of PJI has to be expected,^4-6^ which urges the need to diagnose PJI accurately and promptly for a successful treatment in these patients with a lasting infection-free survival.

Despite the eminent increase in research dealing with the accurate and sensitive diagnosis of hip PJI in recent years, PJI remains a diagnostic challenge.^7-9^ Widely used blood-based tests with serum biomarkers, such as CRP or ESR, may be very limited in detecting low-grade infections in particular, misdiagnosing more than one-third of patients.^7,10-13^ To address these challenges, researchers have explored alternative diagnostic methods such as advanced imaging techniques, molecular assays, and biomarker profiling.^14,15^ Moreover, investigations into the host immune response and identification of specific biomarkers associated with hip PJIs have gained attention as potential diagnostic tools.^16^

Numerous studies have explored the role of microRNA (miRNA) regulation in the immune response against bacterial pathogens.^17-24^ miRNAs are small non-coding RNAs, typically 20 to 22 nucleotides in length, that regulate biological processes including cellular differentiation, development, apoptosis, tumorigenesis, and targeting of foreign pathogens.^24-29^ Due to their stability within biofluids, especially in blood serum,^30^ and their characteristic profiles in various infectious diseases, miRNAs are promising PJI biomarker candidates with extensive diagnostic potential.^31,32^ Currently, there is no study in the literature investigating the diagnostic potential role of miRNA profiling for PJI caused by different virulent microorganisms. The purpose of this pilot study was to analyze different miRNA expressions from blood serum and periprosthetic tissue of patients with PJI of hip arthroplasty, and compare these with patients who had an aseptic revision arthroplasty.

## Methods

### Study design and cohort

The study protocol was reviewed and approved by the institutional ethics committee prior to commencement (EA1/283/19). In this prospective single-centre controlled pilot study, consecutive patients who underwent septic or aseptic revision surgery of hip arthroplasty in our institution (Charité University Hospital, Center for Musculoskeletal Surgery, Berlin, Germany) between November 2021 and July 2022 were enrolled to create three groups (aseptic revision, septic revision due to low-grade PJI, and septic revision due to high-grade infection), each consisting of eight patients. Exclusion criteria comprised concomitant infections of organs other than the ipsilateral hip joint, aged under 18 years, active tumour disease, and antibiotic treatment in the last four weeks preoperatively.

The following data were recorded prospectively for each patient: sex and age, involved joint, clinical symptoms, surgical history of the involved joint, time interval between primary arthroplasty and revision surgery, radiological assessment, and type of revision surgery.

### Standard diagnostic protocol and definition of PJI

The standard diagnostic protocol was established using institutional criteria according to European Bone and Joint Infection Society (EBJIS) to identify PJI.^33^ Infection was classified as low-grade if no signs of acute inflammatory symptoms such as fever, erythema, and warmth were present. The presence of acute signs or symptoms of local inflammation and/or sinus tract with evidence of communication to the joint or visualization of the prosthesis was considered as high-grade infection. The routine PJI protocol at our institution included laboratory values such as serum CRP levels, leucocyte counts, and the proportion of polymorphonuclear leucocytes in aspiration, as well as microbiological and histopathological findings at the time of revision surgery. At least five periprosthetic tissue cultures from various suspicious surgical sites, and at least one specimen for histopathological analysis, in addition to a sonication analysis of retrieved implants, were obtained intraoperatively for each patient. The histopathological analysis was based on the consensus classification of the periprosthetic membrane and neosynovium (formerly called the “synovial-like interface membrane”) presented at the national level in 2004 and at the international level in 2006.^34-37^

Specimens for microbiological analysis were collected with a different, sterile instrument each time and placed immediately into sterile containers without being touched, and analyzed within one hour postoperatively. The microbiological specimens, as well as sonication fluid cultures, were plated onto aerobic and anaerobic sheep blood agar plates and incubated for 14 days. Sonication was performed as previously described.^9^ The number of intraoperative samples and the incidence of positive cultures were documented for each patient. Moreover, radiological and intraoperative evaluation regarding component loosening was recorded. Furthermore, venous blood samples and intraoperative samples from periprosthetic tissue (in patients with PJI, from tissue with punctum maximum of infection) were collected, and plasma and tissue expression levels of different miRNAs were analyzed by small RNA-sequencing (Supplementary Material).^38^

### MiRNA analysis

MiRNA screening and the statistical analysis of preprocessed next-generation sequencing (NGS) data were conducted by TAmiRNA (Austria) using the miRNA NGS discovery (miND) pipeline.^39^ Total RNA was extracted from both plasma and tissue samples using the miRNeasy mini kit (Qiagen, Germany). For plasma samples, the total RNA yield was enhanced by addition of glycogen to the aqueous phase prior to isopropanol precipitation. Total RNAs were eluded in 30 µl nuclease-free water. For tissue samples, RNA yield and integrity was checked using the Bioanalyzer RNA 6000 nano chips (Agilent, USA). For plasma, total RNA concentrations are low, therefore a fixed volume of 8.5 µl was used for library preparation using the RealSeq Biosciences kit. Libraries were quality-controlled and pooled at equimolar rates prior to sequencing on an Illumina Novaseq 6000 SP1 flow cell with 100 cycles (single-end). Fastq files were used for data analysis: following quality filtering, annotation, and filtering of low-abundant miRNAs using the “independent filtering” method provided through DESeq2, differential expression analysis between selected groups was performed using EdgeR with p-value adjustment using the Benjamini-Hochberg method to obtain false-discovery rates (FDRs). A FDR cut-off of 0.05 was applied to identify differentially regulated miRNAs. To visualize miRNA abundance and analyze correlation of miRNAs to clinical data, reads per million (RPM) normalization was applied to correct for variation in sequencing depth.

### Statistical analysis

Patients were trichotomized into three groups depending on the positive culture and grade of the PJI (aseptic vs low-grade vs high-grade). Chi-squared test and Fisher’s exact test were used to find significant differences between categorical variables. For continuous data, Shapiro-Wilk test was performed to assess normality. Analysis of variance (ANOVA) was used to determine if there was a significant difference between the three groups in terms of mean CRP level, mean leucocyte count in aspiration, and mean time interval between primary arthroplasty and revision surgery. The identification of the differences in detail was realized by the Fisher’s least significant difference test. For non-normally distributed values, the Kruskal-Wallis test was applied. Correlation between clinical data (CRP) and microRNA expression values (reads per million) was performed using Spearman correlation and Spearman rank sum test together with Benjamini-Hochberg adjustment, which was applied to assess statistical significance. For statistical analysis, SPSS Statistics v. 29.0 software (IBM, USA) was employed. The results were given as mean and standard deviation (SD) or as number and percentage. A p-value < 0.05 was considered significant in each case.

## Results

### Patient characteristics

The mean age of all 24 patients was 70.0 years (SD 10.2), with 13 being female (54%). PJI characteristics and additional patient demographics are illustrated in Table I. The mean CRP level was significantly higher in the high-grade group compared to the low-grade and aseptic group (147 mg/l (SD 74) vs 11.4 mg/l (SD 12) vs 4.3 mg/l (SD 6), respectively; p < 0.001), as well as the mean leucocyte count in aspiration (150,876 per µl (SD 138,868) vs 15,370 per µl (SD 6,615) vs 766 per µl (SD 770), respectively; p = 0.004). The mean interval between primary arthroplasty and revision surgery did not change significantly between groups (aseptic: 10.1 years (SD 7.4); low-grade: 4.0 years (SD 5.2); high-grade: 8.4 years (SD 8.8); p = 0.251).

**Table I.** Patient demographics and periprosthetic joint infection characteristics.

Sex	Age at revision surgery, yrs	Years between PA and RS	Side	Smoking	Tissue cultures at the time of RS	Sonification microbiology	CRP, mg/l	Histopathology	Surgery for PJI	Aspiration/leucocytes, 1 /nl	Aspiration/PMN, %	Aspiration/microbiology	
**Aseptic**													
Female	85	22.8	R	No	Negative	Negative	5.5	Type 1	Stem exchange	3,146	55	Negative	
Female	57	12.8	R	No	Negative	Negative	0.7	Type 1	One-stage exchange	N/A	N/A	Negative	
Female	68	16.8	R	Yes	Negative	Negative	1.0	Type 1	Head/inlay exchange	807	10	Negative	
Female	88	8.8	L	No	Negative	Negative	18.7	Type 1	Acetabular component exchange	457	55	Negative	
Female	81	6.9	R	No	Negative	Negative	3.8	Type 1	Acetabular component exchange	67	28	Negative	
Female	61	1.0	L	No	Negative	Negative	0.6	N/A	Stem exchange	1,683	57	Negative	
Female	74	9.9	R	No	Negative	Negative	2.2	Type 1	Stem exchange	394	21	Negative	
Male	72	1.7	L	No	Negative	Negative	1.9	Type 1	One-stage exchange	1,957	20	Negative	
**Low-grade**													
Female	70	1.4	L	Yes	*Staph. lugdonensis*, *Staph. hominis*	*Staph. lugdunensis*, *Staph. hominis*	6.5	Type 3	One-stage exchange	15,767	86	*Staph. lugdonensis*	
Male	59	1.1	L	Yes	MRSE	MRSE	8.2	Type 4	One-stage exchange	16,662	95	MSSE	
Male	73	3.4	L	No	Negative	*Finegoldia magna*	14.0	Type 4	Explantation	11,680	91	Negative	
Male	66	16.51	L	Yes	Negative	Negative	40.1	Type 3	Explantation	7,817	82	Negative	
Female	62	0.7	R	No	MSSE, *Staph. hominis*	MSSE	5.7	Type 1	One-stage exchange	N/A	N/A	MSSE	
Male	61	4.5	R	No	MRSE	MRSE	13.2	N/A	Explantation	22,020	83	MRSE	
Male	63	2.3	L	No	Negative	MSSE	0.6	Type 4	One-stage exchange	25,240	24	Negative	
Male	63	2.4	L	No	Negative	*Staph. capitis*	3.4	Type 2	One-stage exchange	8,406	70	*Staph. capitis*	
**High-grade**													
Female	65	13.9	R	No	Negative	Negative	143.5	Type 3	DAIR	36,580	97	*Haemophilus parainfluenzae*	
Male	54	1.9	L	No	*Strept. dysgalactiae*	*Strept. dysgalactiae*	150.8	Type 2	Explantation	N/A	N/A	N/A	
Male	66	1.2	R	No	Negative	Negative	274.0	Type 2	Explantation	9,930	90	Negative	
Female	75	0.9	L	No	*Candida auris*	Negative	66.2	Type 2	DAIR	N/A	N/A	*Candida auris*	
Female	62	24.3	R	No	*Serratia marcescens*	*Serratia marcescens*	55.0	Type 3	Explantation	147,020	73	*Serratia marcescens*	
Male	87	0.1	L	No	*Proteus mirabilis*	*Proteus mirabilis*	194.4	Type 2	DAIR	207,530	86	*Proteus mirabilis*	
Male	89	14.6	R	No	*Strept. angiosus*	Negative	95.4	Type 1	Explantation	353,320	N/A	*Strept. angiosus*	
Female	75	10.6	L	No	MRSE	MRSE	198.6	Type 2	DAIR	N/A	N/A	N/A	

Strept; streptococcus; DAIR, debridement, antibiotics and implant retention; MRSA, methicillin-resistant *Staphylococcus aureus*; MRSE, methicillin-resistant *Staphylococcus epidermidis*; MSSE, methicillin-susceptible *Staphylococcus epidermidis*; N/A, not available; PA, primary arthroplasty; PJI, periprosthetic joint infection; PMN, polymorphonuclear leucocyte; RS, revision surgery; Staph, staphylococcus.

### Circulating miRNA expression profiles

**Fig. 1:**
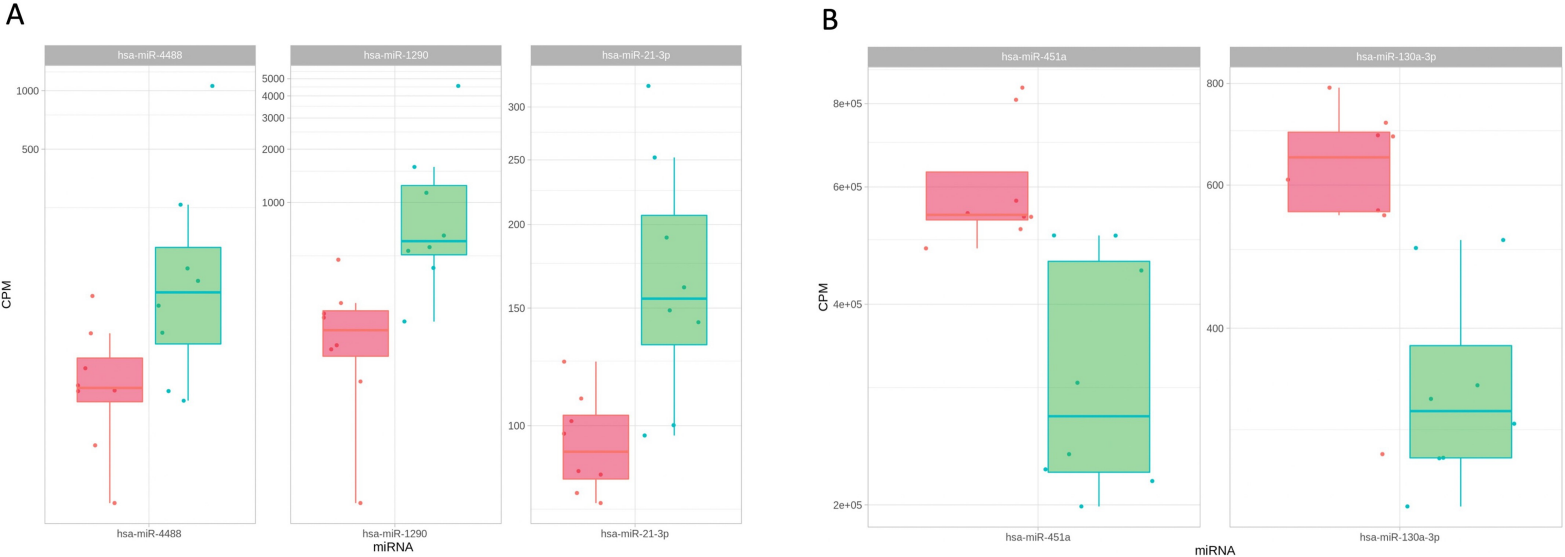
Scatterplots depicting the levels of a) upregulated and b) downregulated micro RNAs (miRNAs) in plasma samples of high-grade (green) vs aseptic (red) group. CPM, counts per million.

**Fig. 2:**
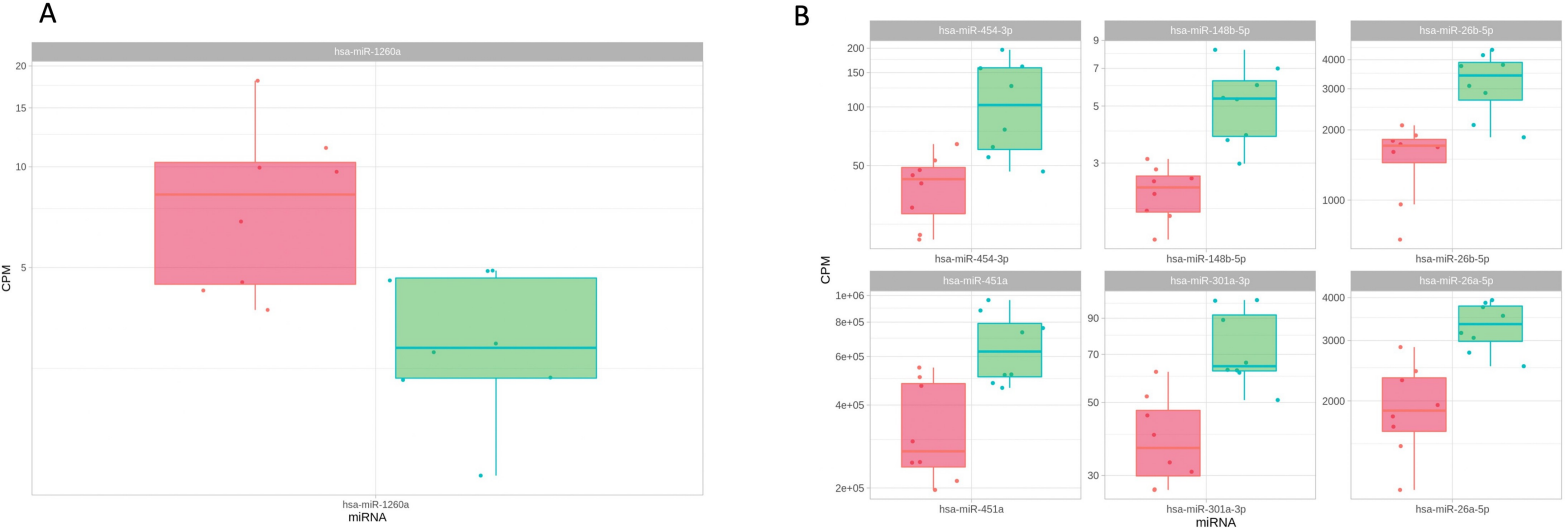
Scatterplots depicting the levels of a) upregulated and b) downregulated micro RNAs (miRNAs) in plasma samples of high- (red) vs low-grade (green) group. CPM, counts per million.

Hsa-miR-21-3p, hsa-miR-1290, and hsa-miR-4488 expression levels were significantly higher in high-grade group compared to the aseptic group (FDR < 0.05) (Figure 1a, Table II). However, results for hsa-miR-130a-3p and hsa-miR-451a also indicated repression of specific miRNA species with high-grade infections compared to aseptic controls (FDR < 0.05) (Figure 1b, Table II). Comparing the high-grade to the low-grade group, we observed lower expression levels of hsa-miR-26a-5p, hsa-miR-26b-5p, hsa-miR-148b-5p, hsa-miR-301a-3p, hsa-miR-451a, and hsa-miR-454-3p in comparison to the low-grade group, whereas a significant upregulation of miRNA could only be observed in hsa-miR-1260a ( p ≤ 0.001) (Figures 2a and 2b). No significant differences were discovered between the low-grade and aseptic groups.

**Table II.** Up- and downregulated micro RNAs in the plasma samples of the high-grade group in comparison to the aseptic group.

miRNA	log2FC	logCPM	F	p-value	FDR
hsa-miR-1290	2.6	9.5	18	< 0.001	0.043
hsa-miR-451a	-0.88	19	15	< 0.001	0.043
hsa-miR-21-3p	0.9	7.1	14	0.001	0.043
hsa-miR-130a-3p	-0.81	8.9	14	0.001	0.043
hsa-miR-4488	2.6	7	13	0.002	0.049

CPM, counts per million; FDR, false discovery rate.

### Circulating miRNAs in correlation with serum CRP levels

A significant correlation existed between serum CRP levels and circulating miRNA dysregulation (Spearman rank sum test adjusted for multiple testing, p.adj < 0.05). In total, 76 different miRNAs in plasma showed a significant correlation with serum CRP levels, of which 75 showed a positive association while only hsa-miR-451a showed a negative association (p.adj = 0.03, *r* = −0.58), indicating that patients with higher CRP levels had more upregulation of these circulating miRNAs. Circulating miRNAs with significant positive association to CRP are illustrated in Table III.

**Table III.** Top ten circulating micro RNAs with significant association to CRP levels.

miRNA	Estimate (r)	p-value	p.adj.
hsa-miR-331-5 p	0.7675	< 0.001	0.002
hsa-miR-589-5 p	0.7299	< 0.001	0.004
hsa-miR-584-5p	0.7286	< 0.001	0.004
hsa-miR-502-3p	0.7182	< 0.001	0.004
hsa-miR-1306-5p	0.7091	< 0.001	0.005
hsa-miR-362-3p	0.6909	< 0.001	0.007
hsa-miR-3605-3p	0.6831	< 0.001	0.008
hsa-miR-454-5p	0.6792	0.001	0.009
hsa-miR-542-3p	0.6792	0.001	0.009
hsa-miR-424-3p	0.6779	0.001	0.009

miRNA, micro RNA.

### miRNA in human tissue samples

**Fig. 3:**
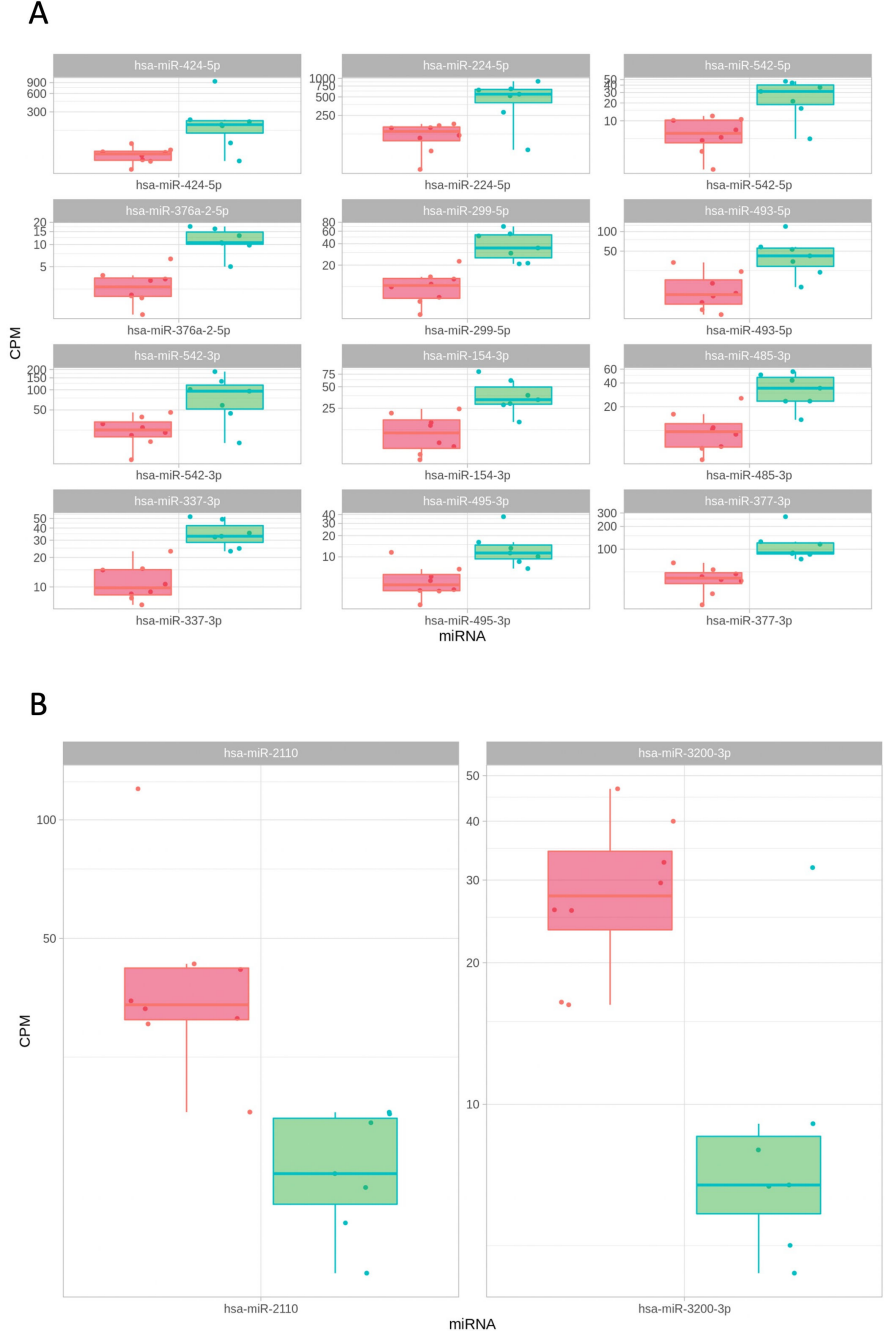
Scatterplots depicting the levels of a) upregulated (top 12 by logFC) and b) downregulated micro RNAs (miRNAs) in tissue samples of high-grade (green) vs aseptic (red) group. CPM, counts per million.

In comparison to the aseptic group, 23 different miRNAs were significantly higher in high-grade group (hsa-miR-154-3p, hsa-miR-224-5p, hsa-miR-299-5p, hsa-miR-329-3p, hsa-miR-370-5p, hsa-miR-376a-2-5p, hsa-miR-376a-5p, hsa-miR-376c-3p, hsa-miR-337-3p, hsa-miR-369-3p, hsa-miR-376a-3p, hsa-miR-377-3p, hsa-miR-382-3p, hsa-miR-424-5p, hsa-miR-485-3p, hsa-miR-487a-3p, hsa-miR-493-5p, hsa-miR-495-3p, hsa-miR-542-3p, hsa-miR-542-5p, hsa-miR-543, hsa-miR-656-3p and hsa-miR-1185-5p; p < 0.05). Only the top 12 miRNA upregulations by log2FC are illustrated in Figure 3a. For hsa-miR-2110 and hsa-miR-3200-3p, expression levels were significantly lower (p ≤ 0.001) (Figure 3b). The dysregulated miRNAs in the tissue samples of the high-grade group compared to the aseptic group are illustrated in Table IV. No significant differences were demonstrated in miRNA expression between high- and low-grade groups, nor between low-grade and aseptic groups.

**Table IV.** Top ten dysregulated micro RNAs in the tissue samples of the high-grade group in comparison to the aseptic group.

miRNA	log2FC	logCPM	F	p-value	FDR
hsa-miR-376a-2-5p	2	2.9	29	< 0.001	0.014
hsa-miR-1185-5p	1.4	4.4	28	< 0.001	0.014
hsa-miR-337-3p	1.6	4.5	26	< 0.001	0.014
hsa-miR-299-5p	1.9	4.6	25	< 0.001	0.014
hsa-miR-377-3p	1.5	6.3	21	< 0.001	0.017
hsa-miR-224-5p	2.1	8.3	21	< 0.001	0.017
hsa-miR-2110	-1.7	4.9	20	< 0.001	0.017
hsa-miR-329-3p	1.3	3	19	< 0.001	0.017
hsa-miR-542-5p	2.1	4.1	19	< 0.001	0.017
hsa-miR-376a-5p	1.4	4.6	19	< 0.001	0.017

CPM, counts per million; FDR, false discovery rate; miRNA, micro RNA.

### Different miRNA expression in plasma and tissues of patients

**Fig. 4:**
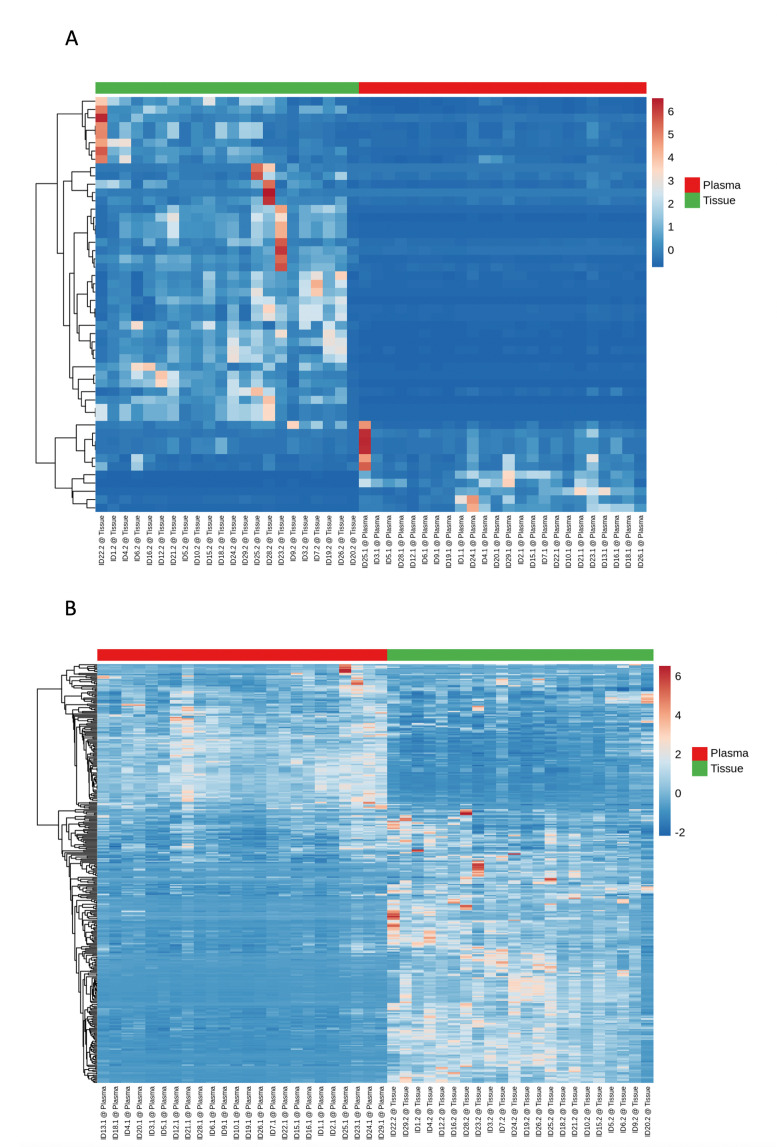
a) This heatmap is for only the top 50 micro RNAs (miRNAs) (based on coefficient of variation (CV%)). An additional filter was introduced to increase the robustness: only miRNAs that showed reads assigned per million (RPM) in at least 1 /n (groups) percent of samples (e.g. with four groups, the miRNA has to have an RPM value above five in at least 25% of the samples). This removed miRNAs that had a high CV, but were only expressed in a too small amount of samples to have any statistical significance or biological relevance. b) A total of 341 miRNAs were shown in the other heatmap, based on the same filters described for the top 50 miRNAs. On the x-axis, plasma and tissue samples are depicted, whereas on the y-axis, the detected miRNAs are illustrated. On the top of the heatmaps, scale bar with plasma (red) and tissue (green) sections is demonstrated.

The present study identified specific miRNAs that were differently expressed in both plasma and tissue samples. The first heatmap (Figure 4a) shows only the top 50 miRNAs. Overall, 341 miRNAs were detected in plasma and tissues (Figure 4b).

## Discussion

To our knowledge, this study represents the first evaluation of miRNA profiling in PJI diagnostics. Currently, approximately 2,600 different miRNAs have been discovered in the human genome, with approximately 2,000 of them identified in the bloodstream circulation.^40^ From this pool of miRNAs, we identified the upregulation of hsa-miR-21-3p, hsa-miR-1260a, hsa-miR-1290, and hsa-miR-4488 in the high-grade group, along with downregulation of hsa-miR-26a-5p, hsa-miR-26b-5p, hsa-miR-130a-3p, hsa-miR-148b-5p, hsa-miR-301a-3p, hsa-miR-451a, and hsa-miR-454-3p compared to the aseptic and low-grade groups. Some of these miRNA dysregulations have been described in other contexts in recent literature. For instance, miR-21, well established as an oncogenic miRNA,^41^ regulates phagocytosis during infection, potentially limiting the availability of the intracellular niche of pathogens like *Listeria monocytogenes*.^42^ Hsa-miR-130a is relevant in the colonization of intestinal mucosa in Crohn’s disease patients, facilitating the intracellular replication of adherent–invasive *Escherichia coli.*^43,44^ Hsa-miR-451, one of the miRNAs controlling the NF-kB pathway, is upregulated during chlamydia infection.^45^ However, hsa-miR-451-upregulation has also been associated with haemolysis, a potential confounder of circulating miRNA data.^46,47^ Other miRNA dysregulations identified in this study were demonstrated for the first time in the literature in the context of a bacterial infection. This suggests a crucial step in establishing miRNA signatures as potential novel diagnostical tools and prognostic serum biomarkers for hip PJI.

In the present study, high-grade PJI showed significantly higher expression levels for 23 different miRNAs and lower expression levels for hsa-miR-2110 and hsa-miR-3200-3p in tissue samples compared to patients with no infection. To our knowledge, these miRNA signatures were described for the first time in accordance with bacterial infection, which is a crucial first step for tissue miRNA profiling in the diagnosis of hip PJI.

The present study revealed that miRNA profiles expressed in tissue vary from those in plasma. This incongruence between the miRNA expression profiles suggests that miRNAs do not simply “leak” from infected tissue, as the plasma profile does not simply reflect the tissue profile. This observation is highly indicative of differential pathological processes occurring between plasma and tissue cells. The characteristic distribution of extracellular miRNAs suggests that they may constitute a specific biological response. This unexpected level of complexity needs to be considered with regard to the use of serum biomarkers with miRNA profiling of plasma and tissues for diagnosing hip PJI.

Among the miRNAs circulating in the bloodstream and correlating with CRP serum levels, the let-7 family is one of the most extensively researched miRNAs in relation to infection.^24^ It is associated with cell differentiation and development,^48^ and functions as an actor of the acute innate immune response.^22^ The present study detected an upregulation of the let-7 family in correlation to CRP, except for hsa-let-7d-5p, which was downregulated upon CRP increase. Moreover, some of the detected upregulated miRNAs in correlation with CRP increase such as hsa-miR-127, hsa-miR-193, hsa-miR-212-3p, hsa-miR-365, and hsa-miR-432-5p were already described in literature in the context of bacterial infection.^49-51^

MiRNAs function as important regulators in bacterial infection, suggesting their great application potential as novel therapeutic targets to cure bacterial infections. Although there are no bacterial infection-related miRNA-based drugs investigated in clinical research to date, miRNAs still represent a promising approach for future therapies or as immune modulators against invading pathogens. It is important to note that detected regulations of miRNAs in the high-grade group compared to the aseptic group were irrespective of bacterial pathogens. For this reason, it can be concluded that miRNA expression during infection is not specific to the bacterial pathogen but rather to the reaction of the host against it. Moreover, individual genes are usually not controlled by a single miRNA and the ramifications of the identified miRNA species on the severity of PJI need to be further evaluated. The regulation of the miRNAs illustrated in this study may influence different molecular biological processes in the development and progression of PJI, representing potential targets in therapy.

This study has several limitations. First, our study contains a small sample size, which is possibly why we could not identify any significant difference in miRNA dysregulation between aseptic and low-grade groups in plasma and tissue samples, and between low- and high-grade groups in tissue samples. Second, the present study is neither randomized nor blinded, inevitably introducing selection and evaluation biases. Additional double-blind randomized studies, including a larger cohort number, are required to further substantiate these findings. Moreover, low-grade infections may not have the virulence to induce significant differences in miRNA expression. Nevertheless, we were able to identify circulating miRNA signatures and local expression profiles in tissue and blood samples, correlating with the severity of hip PJI for the first time. Third, including another group of patients with primary septic arthritis of the hip would have been beneficial to demonstrate the specificity of the identified miRNAs for implant-related infections. Lastly, there is no consensus on the the PJI definition criteria. None of the existing definition criteria are uniformly accepted and may largely influence the clinical course of the patients as well as the outcomes. However, the EBJIS criteria demonstrate a high sensitivity for low-grade PJIs as reported in literature.^12,33^

High-grade hip PJI correlates with individual plasma and tissue miRNA signatures compared to aseptic patients with the revision of hip arthroplasty. The present study illustrates different miRNA expressions, contributing to the pool of miRNA signatures as potential novel diagnostic and prognostic biomarkers for hip PJI.

**Take home message**

- With this prospective pilot study, we were able to identify a circulating microRNA (miRNA) signature correlating with high-grade periprosthetic joint infection (PJI) compared to aseptic patients undergoing hip arthroplasty revision.

- Our data contribute to establishing miRNA signatures as potential novel diagnostic and prognostic biomarkers for PJI.

## Data Availability

All data generated or analyzed during this study are included in the published article and/or in the supplementary material.
